# Implementation of a vaccine against Shigatoxin 2e in a piglet producing farm with problems of Oedema disease: case study

**DOI:** 10.1186/2055-5660-1-6

**Published:** 2015-05-07

**Authors:** Regine Fricke, Olaf Bastert, Verena Gotter, Nico Brons, Johan Kamp, Hans-Joachim Selbitz

**Affiliations:** 3 IDT Biologika GmbH, Am Pharmapark, 06861 Dessau-Rosslau, Germany; 4 Dierenkliniek Hellendorn, Ommerweg 54, 7447 RG Hellendoorn-Nijverdal, Kragujevac, Netherlands

**Keywords:** Oedema disease, Vaccine, Reduction of antimicrobial use, STEC, EDEC, Shigatoxin 2e

## Abstract

Oedema disease is one of the major diseases in pigs during the nursery period. It is caused by Shigatoxin 2e producing strains of *Escherichia coli*. In order to combat the disease, the metaphylactic use of colistin sulphate and zinc oxide is widely spread. Additionally, special feeding regimens such as the reduction of the amount of crude protein and the increase of the amount of crude fibre are applied. The goal of this study was to test the efficacy of a vaccine against Oedema disease caused by Shigatoxin 2e in a field trial on a farm with a history of Oedema disease in nursery pigs.

The study was carried out on a Dutch farm with 600 sows and a one-week farrowing rhythm and lasted for the time of one year. During this time all piglets were vaccinated with 1 ml ECOPORC SHIGA at the average age of 4 days. The parameters Overall mortality, use of antimicrobials in general, calculated as defined daily dose per animal, use of colistin sulphate and the weight gain were evaluated for all nursery pigs and compared to historical data of animals from the same period of time directly prior to the study serving as a historical control group.

The previous mortality in the nursery of 7.7% was significantly reduced to 1.3% after vaccination. The metaphylactic use of colistin sulphate during the nursery period was stopped during the study because no deaths due to Oedema disease had occurred anymore after beginning of vaccination. The defined daily dose per animal per month was significantly reduced from a mean of 1.050 in the year 2012 to a mean of 0.215 in the year 2013. The defined daily dose per animal per year was therefore relevantly reduced from 12.6 in 2012 to 2.6 in 2013.

These results show that on this farm Oedema disease can not only be controlled successfully by vaccination but also that vaccination can significantly reduce the use of antimicrobials in the nursery period.

## Background

Oedema disease (ED) is one of the most costly diseases in pig production today. It is caused by Shigatoxin 2e (Stx2e) producing strains of *Escherichia coli* (STEC), which are sometimes also called Verotoxin producing *Escherichia coli* (VTEC) [1]. Those STEC strains that cause Oedema disease are called (EDEC). For many years, the only way to fight the disease was by a combination of the metaphylactic use of antimicrobials such as colistin sulphate and/or zinc oxide and diverse management practices. However, the widespread use of antimicrobials in livestock production is increasingly being questioned by the public, also because the use of antimicrobials [2] and zinc oxide increases resistance in *E. coli* [3]. While in general the resistance of *E. coli* to colistin is low (<1%), in some cases in Belgium it reached 9.5% in sick pigs [2]. Therefore many governments in Europe (for example Denmark, Germany and the Netherlands) have passed legislation to reduce the use of antimicrobials. In the Netherlands for example a benchmark system classifies farms into 3 different categories according to their consumption of antimicrobials calculated as Defined Daily Dose per year (DDD/y) on farm level. A classification into a category with high antibiotic use leads to requirements to reduce the use of antimicrobials [4].

Among the diverse management practices developed to prevent the occurrence of ED, the most popular is to adjust the feeding regimen of the piglets after weaning, as this is implemented most easily [5]. The adjustment mainly consists of reducing the amount of crude protein in the diet from 210 g/kg to at least 180 g/kg while at the same time increasing the amount of crude fibre from 30–50 g/kg to 60–65 g/kg. However this method alone is often not enough to reduce the occurrence of ED and antimicrobials still have to be used. Also it has been theorized that due to this type of feeding regimen, piglets cannot gain the weight that their genetic potential would offer [6].

Because the most common adhesion factor by which EDEC bind to the enterocytes in the gut are the F18 fimbriae [1] geneticists have been trying to breed a F18-resistant pig for some years [7]. This may be an effective method to face the disease. Still most pigs though are susceptible to F18 bearing *E. coli*.

Research groups around the world also used the Stx2e itself as a focal point, primarily when looking for vaccines against ED. Recombinantly produced and by glutaraldehyde inactivated Stx2e was tested successfully in field trials with pigs [8–13]. In each case mortality was significantly lower in the vaccinated groups and partially a significantly higher weight gain of the vaccinated animals could be shown. In laboratory trials recombinantly produced and genetically inactivated Stx2e proved to induce protection against an oral challenge with EDEC [14] as well as an intravenous challenge with Stx2e [15]. Mortality and clinical signs of ED could be prevented (0%) whereas the control animals became severely ill (clinical signs 59% resp. 100%, mortality 35% resp. 33%). Within laboratory trials for the recombinantly produced and genetically inactivated vaccine ECOPORC SHIGA, which was subject to this investigation, a significant protection against ED could be reached by vaccination [16]. Furthermore, the first field trials for the investigation of safety and efficacy of ECOPORC SHIGA conducted on farms affected by ED showed a significantly reduced morbidity and mortality as well as a higher weight gain for the vaccinated groups [17–19]. First large-scale longitudinal studies with data from historical control groups (HCG) within Germany [20–22] demonstrated that the mortality due to ED as well as the use of colistin sulphate were significantly reduced by vaccination with ECOPORC SHIGA compared to a former period without vaccination. Furthermore the weight gain was partially increased in these studies [20]. Vaccination against the disease seems a good alternative to antibiotic treatment, because there is no risk of the development of resistances in *E. coli.* Additionally, no special feeding is required.

The goal of this case study was to investigate the ability of the commercially available vaccine ECOPORC SHIGA of preventing ED on a Dutch farm with a high mortality despite the use of colistin sulphate.

## Case description

### Case history

A 600 sow farm (breed: Topigs) in the Netherlands had massive problems with ED in the nursery for over a year. The farm is a single site facility with the farrowing barn separated from the nursery. The farrowing rhythm is one-week. Cross fostering is carried out until the age of 3 days but is kept to a minimum in order to avoid the transmission of *Streptococcus suis*. Sows are vaccinated against Parvovirus, Swine Erysipelas, Swine Influenza and Colibacillosis in neonatal piglets. Weaning takes place at an age of 25–26 days when the piglets weigh approximately 7.5 kg. After weaning, the piglets are transferred to the nursery. The nursery is run all in – all out by compartment. Each age group has one compartment and age groups are not mixed. Space and feeding requirements are met according to law. Water is supplied via drinking nipples ad libitum. Nursery period ends at the age of 11 – 11.5 weeks.

Within the period between 01.10.2011 and 30.09.2012 the mortality in the nursery was at 7.7% with a confirmed mortality due to ED of 5.9%. Implemented measures like low crude protein content of 160 g/kg in the feed as well as the metaphylactic treatment of each weaned week group with Colistin sulphate for the first 10 days in nursery did not have any valuable impact on reduction of mortality. The diagnosis of ED had been made based on the clinical examination of affected piglets as well as the results of necropsies and microbiological analysis.

### Study description

For the study a total of 17977 piglets were included and vaccinated within the period from 02.10.2012 to 24.09.2013. Inclusion criteria were: a weight of more than 800 grams, no clinical signs of an illness and the clear assignment to a specific sow. Piglets that did not meet these criteria were not vaccinated and the number of their respective ear tags was registered.

The piglets were vaccinated intramuscularly at the base of the ear with a dose of one millilitre containing a genetically modified and recombinantly produced Stx2e (trade name ECOPORC SHIGA) at the average age of four days. One dose of the vaccine (1 ml) contains ≥ 3.2 × 106 ELISA units of recombinant Stx2e. The adjuvant is aluminium hydroxide. At the same time, the piglets were ear tagged with different coloured tags for each week group, as well as being injected with iron (other side of the neck), the tails were docked and male piglets were castrated.

Every day during the nursery period, the farmer observed whether the piglets showed any clinical signs of ED (Oedema of the eyelids and subcutis of the face; nervous disorders like ataxia, paralysis of limbs, tonic-clonic convulsions) and controlled the general appearance of the piglets. When piglets showed signs of illness, a clinical diagnosis was made whether or not the occurring clinical symptoms were related to Oedema disease. In case of losses for which the farmer was not able to define a reason these piglets were sent for pathological examination to examine the definite cause of death. This included special investigations concerning the appearance of Oedema disease, like PCR-testing of the isolated *E. coli* strains regarding factors of pathogenicity and histopathological examination of e.g. the brain. In order to assure the presence of EDEC despite vaccination rectal swabs were taken from vaccinated 7 weeks old animals by the herd attending veterinarian at the end of the study. No changes were made regarding the content of crude protein in the feed during the study in order to maintain equality of the requirements for comparison.

Observed and compared data were the Overall mortality (expressed as the mean percentage of all dead piglets during nursery period per weaned piglets on monthly basis without regard to the reason of death), the metaphylactic use of colistin sulphate (defined as DDD animal colistin sulphate/month) and the general use of antimicrobials (defined as Mean DDD animal/month) within the period of one year prior to vaccination and one year with vaccination. Additionally, the mean body weight at sale of the total of pigs sold in the defined period was calculated via software (Pigmanager) and compared to the previous period with unvaccinated animals. The vaccination was performed by the herd attending veterinarian. The observations and recording of the data were carried out by the farmer, following training in the study protocol and was supervised by the herd attending veterinarian. Statistical analyses were performed by SPSS 15.0 for Windows (SPSS Inc., Chicago, Illinois, USA). Analyses performed included the Mann–Whitney-U-test.

Ethical approval for this study was granted by our company IDT, in line with requirements in Germany for studies involving animals. The proper authorities in the Netherlands were also notified prior to the start of the study, and granted all required approvals.

### Results

The mean Overall mortality of 7.7% prior to vaccination was significantly reduced to 1.3% after vaccination (p < 0.001, Mann–Whitney-U-test, two-tailed, asymptotic, see Table 1).

**Table 1:** **Overall mortality during nursery period**

Group	Overall mortality during nursery period (%)	std dev	N	p-value
Pretrial	7.7	2.70	12	<0.001
During trial (ECOPORC SHIGA)	1.3	0.54	12	

N = Number of months recorded.

One piglet of the 11^th^ vaccination group was classified as “death due to Oedema disease”, but only by default. When this piglet died, it was failed to be submitted to necropsy and subsequent analysis. Thus because the cause of death could not be determined, it was decided to classify its death as due to Oedema disease. In two animals clinical signs associated with ED (recumbence with tonic-clonic convulsions) were observed after weaning. After the piglets had died pathological examinations as well as microbiological analyses were carried out. *Streptococcus suis* was determined to be the cause of the clinical signs. The analysis for Stx2e producing *E. coli* of rectal swabs taken from 7 weeks old pigs at the end of the study showed that the pathogen was still present on the farm despite vaccination.

The metaphylactic use of colistin sulphate was stopped after the 9^th^ weaned group of vaccinated animals, because until then no more deaths due to ED had occurred. It was not implemented anymore during the study period. The defined daily dose per animal (Mean DDD animal/month) for all antimicrobials was reduced from a mean of 1.050 in the year 2012 to a mean of 0.215 in the year 2013 (p < 0.001, Mann–Whitney-U-test, two-tailed, asymptotic, see Table 2).

**Table 2:** **Defined daily dose per animal (DDDanimal) per month**

Year	Mean DDDanimal/month	std dev	N	p-value
2012	1.050	0.479	12	<0.001
2013	0.215	0.217	12	

N = Number of months recorded.

The sum of the Mean DDD animal/month for the year 2012 was 12.6. This was reduced to 2.6 for the year 2013. The mean body weight of the sold groups was higher after vaccination (25.252 kg vs. 25.618 kg respectively), but this difference was not statistically significant.

## Discussion and evaluation

In this study the effect of vaccination on the Overall mortality was evaluated instead of the effect on mortality only due to ED. This was due to the long period of time of the study so that not every dead piglet could be sent for necropsy in order to define the definite cause of death. The assumption was made that if vaccination had an effect on mortality due to ED then the Overall mortality would be reduced significantly in comparison to the Overall mortality within the same period of time without vaccination. With the high mortality due to ED on the farm and therefore ethical reasons a HCG was chosen for the comparison of data instead of a randomised control group (RCG). Although the choice of a HCG has the potential to bias a study as different conditions might be existent this was thought to be justifiable, considering that not just any period of time for the historical comparison was chosen but exactly the same time frame of 1 year. That time frame was set exactly prior to the start of vaccination so that both comparison groups were directly adjacent to each other. Furthermore all animals from that previous period were included for the HCG and not only a selected group of animals. These measures were supposed to minimize any impact of potential different conditions as far as possible. In order to maintain comparability between the HCG and the vaccinated study group regarding the influence of ED the implemented control measures like dietary crude protein content and initially the treatment with colistin sulphate were not changed. Thus the influence of vaccination only was supposed to be investigated. As the use of antimicrobials was a parameter to be compared between the groups and due to the fact that after vaccination no deaths related to ED had occurred anymore this control measure was ceased. On the other hand though, the impact of a higher content of crude protein in the diet could neither be evaluated on the weight gain nor on the development of the disease on farm level. Furthermore the presence of EDEC in vaccinated animals was ensured by examination of rectal swabs. This was expected and is due to the fact that the vaccine induces protection against the Stx2e in the blood but not against EDEC in the gut. In order to ensure a consistent elicitation of the comparative parameters the study data was collected in the same way as the historical data: The mortality had been recorded by the farmer and additionally monitored by the herd attending veterinarian within the framework of the integrated veterinary herd health care. Body weight at sale had been collected routinely by suitable computer software (Pigmanager) and the data on antibiotic use und thus the DDD had been recorded by an official registration system.

The significant reduction of the Overall mortality by 6.4% confirms not only the results from other investigations with RCG’s in which ECOPORC SHIGA significantly reduced the mortality on farms with ED [17–19] but also results from other longitudinal trials with HCG’s and with high numbers of animals [20–22]. The reduction resp. omission of colistin sulphate led to a significant reduction of the DDD during the nursery period so that the DDD on farm level was significantly reduced and the farm was classified into a better category of the Dutch benchmark system. The long lasting reduction of antimicrobial use after implementation of the vaccination could also be seen in other large-scale longitudinal studies [20–22] and responds to the requirements of a reduced use of antimicrobials in animal husbandry.

Due to the application of the vaccine at the age of four days, it was easy for this farmer to integrate the measure into his routine handling of the piglets. Since in modern pig production zoo-technical measures are often performed at the age of three to seven days, the ability of vaccination from the age of four days on makes it possible for such farms to include this vaccination into their routine. Also the application dose of one millilitre is small, an additional advantage when dealing with young piglets.

## Conclusions

From the results of this study it may be concluded that the vaccination against Stx2e led to a significant reduction of mortality due to ED on this farm. Also it appears likely that the implementation of the vaccination on this farm has significantly reduced the used amount of antimicrobials during the nursery period. These results confirm similar experiences made with this vaccine on other farms with ED in other trials.

## Abbreviations

DDD: Defined daily dose
ED: Oedema disease
F18: Adhesion factor of *Escherichia coli*
g/kg: Grams per kilogram
kg: kilograms
STEC: Shigatoxin producing *Escherichia coli*
VTEC: Vero-toxin producing *Escherichia coli*.
